# Effects of concurrent training sequence on VO_2max_ and lower limb strength performance: A systematic review and meta-analysis

**DOI:** 10.3389/fphys.2023.1072679

**Published:** 2023-01-26

**Authors:** Jiuxiang Gao, Liang Yu

**Affiliations:** ^1^ Laboratory of Exercise Physiology, College of Sports Science, Beijing Sport University, Beijing, China; ^2^ Laboratory of Fitness Training, College of Fitness Training, Beijing Sport University, Beijing, China

**Keywords:** concurrent training, training sequence, endurance training, strength training, VO2_max_, lower limb strength

## Abstract

The aim of this study is to compare the effects of concurrent strength and endurance training sequences on VO_2max_ and lower limb strength performance to provide scientific guidance for training practice. We searched PubMed, EBSCO, Web of Science (WOS), Wanfang, and China National Knowledge Infrastructure (CNKI) databases up to December 2022. The included articles were randomized controlled trials that allowed us to compare the strength–endurance (S-E) sequence and endurance–strength (E-S) sequence on VO_2max_, maximum knee extension strength, maximum knee flexion strength, and lower limb power. The Cochrane bias risk tool was used to evaluate the methodological quality of the included literature, and Stata 12.0 was used for the heterogeneity test, subgroup analysis, draw forest map, sensitivity analysis, and publication bias evaluation. The results have been presented as standardized mean differences (SMDs) between treatments with 95% confidence intervals and calculations performed using random effects models. Significance was accepted when *p* < 0.05. The studies included 19 randomized controlled trials (285 males and 197 females), 242 subjects in S-E sequence, and 240 subjects in E-S sequence in the analyses. No difference changes between S-E and E-S sequences has been observed on VO_2max_ in the overall analysis (SMD = 0.02, 95% CI: −0.21–0.25, *p* = 0.859). The S-E sequence shows a greater increase in lower limb strength performance than does the E-S sequence (SMD = 0.19, 95% CI: 0.02–0.37, *p* = 0.032), which was manifested in the elderly (*p* = 0.039) and women (*p* = 0.017); in training periods >8 weeks (*p* = 0.002) and training frequencies twice a week (*p* = 0.003); and with maximum knee flexion (*p* = 0.040) and knee extension strength (*p* = 0.026), while no difference was found in lower limb power (*p* = 0.523). In conclusion, the effect of VO_2max_ will not change with different concurrent training sequences. The S-E sequence improves lower limb strength more significantly, mainly in the improvement of knee flexion and knee extension. This advantage is more related to factors such as age, gender, training period, and training frequency.

## 1 Introduction

Indeed, high levels of muscular strength and aerobic endurance are the key determinants of success in many sports. For example, rowing is a typical hybrid endurance–strength sport. Also, in a single hockey or football game, it is very important for athletes to deliver a hard body confrontation (strength and hypertrophy) or accelerate suddenly to get rid of the defender (power) and persevere long-time running (endurance). A promising way to increase performance is to train both muscle strength and cardiorespiratory fitness within a training cycle. The inclusion of resistance training (to gain strength, hypertrophy, and power) combined with aerobic exercising (to enhance endurance) in a single program is known as concurrent training. It is a popular training strategy to develop various aspects of physiological capabilities, potentiate the individual effects produced by endurance training and strength training, and increase motor performance more than training alone.

However, with the increase of endurance training, muscle strength, hypertrophy, and power will decline during concurrent training. Hickson (1980) first proposed that the interference of concurrent training shows that strength performance is negatively affected by endurance training when compared to it being performed alone, and this is due to differences in the physiological competing adaptation of muscles to strength and endurance training over a long-term training program. Strength training generally increases muscle fiber recruitment, ATP-CP, and glycolytic enzyme activity and exhibits increased muscle cross-sectional area and strength, but it decreases mitochondrial and capillary density and number within the muscles. Endurance training improves the oxygen utilization capacity of muscles by increasing cardiopulmonary function, myoglobin number, mitochondrial density and number, and aerobic enzyme activity, but it is accompanied by a decrease in the cross-sectional area of muscle fibers, which reduces the level of muscle strength or power (Glowacki et al., 2004). In addition, it has been shown that exercise mode, period, frequency, and intensity and subject characteristics may influence the outcome of concurrent strength and endurance training (Docherty and Sporer, 2000; Fyfe et al., 2014). However, it is not well known whether the sequence of strength and endurance training in a concurrent training session impacts chronic adaptations.

In a training sequence, Makhlouf et al. (2016) found that the strength prior to endurance training could improve the dynamic strength of muscles more than the opposite training sequence, which may be related to endurance training, leading to fatigue that affects neuromuscular activation and reduces muscle firing frequency. Doma and Deakin (2013) reported that long distance runners have more advantages in improving performance and running economy after endurance training prior to strength training, with the sessions separated by 6 h. Moreover, 12 weeks of strength training after high-intensity intervals training (HIIT) can better improve 4-km running performance and VO_2max_ than the reverse training sequence (Chtara et al., 2005). Wilhelm et al. (2014) found that regardless of the sequence of endurance and strength training, it was beneficial to strength and power output in older adults. In addition, there are reports that athletes at different training levels have certain differences in the sequence of endurance and strength training (Chtara et al., 2008). Current meta-analyses related to concurrent training sequences remain questionable as to whether the training methods, training periods, and training frequencies promote greater endurance and strength performance (Murlasits et al., 2018). It can be seen that current studies have contradictions and only few systematic studies have been carried out on the benefits of concurrent training sequences for solving the needs of training practices and improving training levels.

The maximal oxygen uptake (VO_2max_) represents the limit value of aerobic exercising and is one of the important indicators for evaluating aerobic capacity. One-repetition maximum (1RM) is considered to be the basis of strength ability, and extensive studies have confirmed that performances such as long jump and vertical jump are highly correlated with lower extremity muscle strength (Castro-piñero et al., 2010). These indicators can be evaluated as endurance and strength abilities. Accordingly, we have systematically collected and screened studies on concurrent training sequences and demonstrated the effect of concurrent training sequences on VO_2max_ and lower body strength–related indicators such as age, gender, training time, training frequency, and training methods. Our hypothesis is that different concurrent training sequences would affect the lower limb strength performance in various ways. However, due to data constraints, the proportion of endurance and strength training cannot be obtained. This study cannot provide the dominant position of endurance and strength training.

## 2 Methods

### 2.1 Experimental approach to problem

To test our hypothesis, we performed a systematic review through meta-analyses of longitudinal studies, investigating the effect of concurrent training sequences on VO_2max_ and lower limb strength performance. The eligibility criteria were established, and the systematic review was registered at http://www.crd.york.ac.uk/prospero as CRD42022306083. This study has been reported according to the Preferred Reporting Items for Systematic Reviews and Meta-Analyses (PRISMA).

### 2.2 Procedures

#### 2.2.1 Eligibility criteria

The specific inclusion criteria were (a) research objects: different age groups that received both endurance and strength training during the same session; (b) intervention and control measures: one group received the method of strength training before that of endurance training (S-E), and another group received the method of endurance training before that of strength training (E-S); (c) outcome indicators: lower limb muscle strength 1RM (leg press, leg curl, and knee extension), squat jump (counter movement jump, CMJ), and VO_2max_; and (d) research design: randomized controlled experiment. Research studies were excluded if they were (a) literature that did not meet the requirements of the previous inclusion criteria; (b) non-Chinese, English, and non–full text documents; (c) studies with animals; (d) published reviews, conference communications, opinion articles, commentaries, book chapters, case studies, or presentations; (e) training interventions not related to the order of endurance and strength training; and (f) one-time training intervention or training that contained training contents other than endurance and strength training.

#### 2.2.2 Search strategy

A comprehensive database search was systematically conducted using PubMed, EBSCO, Web of Science (WOS), Wanfang, and China National Knowledge Infrastructure (CNKI) databases up to 20 December 2022. In addition, two authors (Z.B.Z. and J.C) also conducted a manual search of the references included in the study to ensure that all relevant studies were captured. The search language included English and Chinese, and the strategy keywords included variations on terms related to “concurrent training,” “training sequence,” “VO_2max_,” and “lower body performance.” To optimize the capture of relevant references, such terms were combined by Boolean operators (OR and AND). The complete retrieval process took PubMed as an example, and the retrieval scope was [All fields]. #1: (concurrent) OR (combined) OR (combination); #2: (endurance training) OR (aerobic training); #3: (strength training) OR (resistance training); #4: (training sequence) OR (training order); #5: (VO_2max_) OR (aerobic capacity) OR (RM) OR (repetition maximum) OR (muscle strength) OR (muscle power) OR (lower limb performance) OR (lower limb strength) OR (lower limb power) OR (leg curl) OR (knee extension) OR (CMJ) OR (jump) OR (aerobic performance); and #6: #1AND#2 AND#3 AND#4 AND#5.

#### 2.2.3 Selection of studies

The selection of studies was based on the eligibility criteria adopted and performed independently and in duplicate. First, two authors (Z.B.Z. and J.C) evaluated the titles and abstracts of all studies found from the search. Articles whose abstracts did not provide sufficient information as per the inclusion and exclusion criteria were assessed separately in full. The list of retrieved articles was screened independently by two authors (Z.B.Z and J.C) to choose potentially relevant articles. Disagreements were resolved by consensus, and in case of perseverance, a third researcher (Y.G) resolved their differences.

**FIGURE 1:**
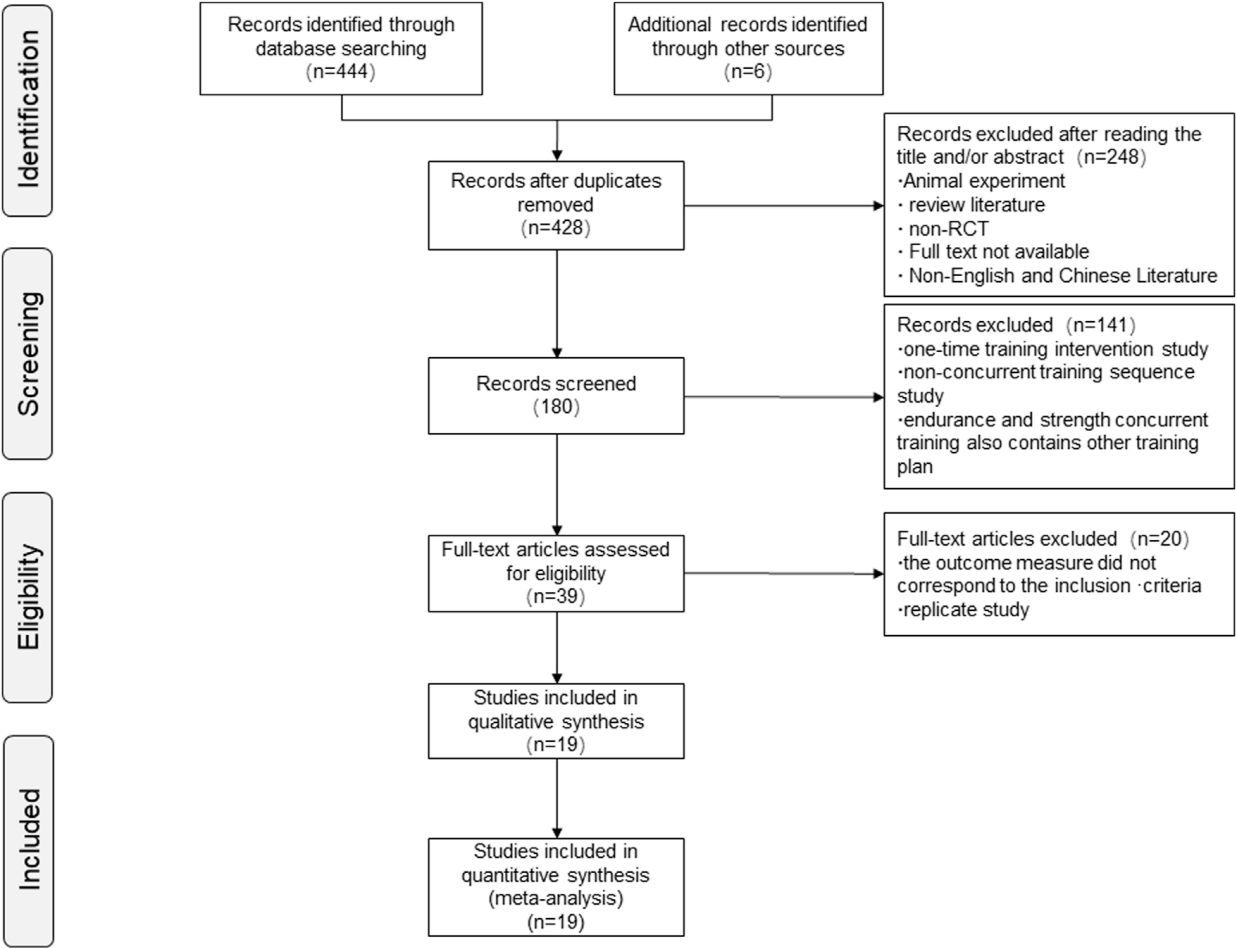
Flowchart of studies included.

Through the literature retrieval strategy, we obtained 444 articles from the databases and six articles from other sources related to concurrent training sequence, VO_2max_, and lower limb strength performance. After removing duplicates, 428 articles were screened for eligibility on the basis of their title and abstract, with 248 being subsequently excluded. In the primary selection of 180 articles, 141 articles were excluded from the one-time training intervention study, non–concurrent training sequence study, and endurance and strength concurrent training those contained other training plans. A total of 39 studies were assessed as full texts, and 19 studies (Figure 1) were included in the qualitative analyses (meta-analysis). From these, 20 studies were excluded because variables did not correspond to the final outcome variables analyzed (VO_2max_; lower body repetition maximum such as leg press, leg curl, knee extensors, CMJ, and five jumps).

#### 2.2.4 Data extraction

From the included studies, three authors (J.X.G, Z.B.Z, and J.C) extracted to a previously designed data sheet the following collected information: author, year of publication, study subjects, gender, age, sample size, training period, training frequency, basic information, and training program. Missing data were requested from the researcher of the study in question; in case of no answer, denying provision, or data loss, the article or outcome was excluded. For data presented only graphically, the results were extracted using the GetData Graph Digitizer software.

The outcome variables for aerobic capacity were VO_2max_; the methods of the VO_2max_ test that included a progressive exercise test, Bruce protocol on treadmill, and 20 m shuttle run; lower body repetition maximum such as leg press, leg curl, and knee extensors; and lower body power such as CMJ and five jumps. Some authors were contacted to complete missing data regarding the main outcomes. The extracted outcomes were the absolute deltas of the values. When not available, the delta was calculated from the values obtained before and after the intervention, and the delta SD was imputed by the equation proposed by Higgins and Green (2008).

#### 2.2.5 Risk of bias assessment

The assessment of risk of the bias tool was carried out in accordance with the Cochrane Handbook 5.1.0. The individual studies included adequate random sequence generation, allocation concealment, blinding of participants and personnel, blinding of outcome assessment, incomplete outcome data, selective reporting, and other bias.

When these characteristics were described in the published document, it was considered that the criteria were met, and they were classified as “low risk” or “high risk.” Studies that did not describe these data were classified as “unclear risk.” This evaluation was performed independently by two groups of reviewers (J.X.G/Y.G. and Z.B.Z/J.C.).

### 2.3 Statistical analyses

The literature screening chart and Cochrane bias risk assessment diagram were made using the Review Manager software 5.4. The Stata 12.0 software was used to perform the heterogeneity test, data merging, subgroup analysis, forest plot, and sensitivity analysis (metainf and galbr tests) for the outcome indicators of the included literature. Publication bias was verified through the Begg's test and Egger's test and was considered to be significant when *p* < 0.10 (Eng et al., 2014). In case of publication bias, the trim-and-fill test was used to estimate the effects of publication bias on interpreting the results.

Since the outcome indicators of the included literature are continuous outcome variables, when evaluating lower extremity muscle strength due to the different units of the selected indicators or when evaluating VO_2max_ and because the experimental data of Eklund et al. (2016) are expressed as a percentage, the results are presented as standardized mean differences (SMDs) between treatments with 95% confidence intervals (CIs).Heterogeneity between studies was evaluated through I^2^, where a value of <25% was assessed as low magnitude, between ≥25% and ≤75% was set as medium magnitude, and >75% was considered high magnitude. The calculations were performed using the random effects model when the heterogeneity was obvious (I^2^ > 50%); otherwise, the fixed effects model was used. The values of *p* ≤ 0.05 were considered statistically significant; the very significant level was *p* < 0.01.

Statistical analyses were performed through a meta-analysis comprising the comparison of S-E with E-S training sequence on VO_2max_ and lower body performance. Subgroup analyses included comparisons between the S-E and E-S groups with different age, gender, training frequency, training period, and training models. In addition, sensitivity analyses were performed for lower body performance.

## 3 Results

### 3.1 Description of studies

Subjects and study characteristics are summarized in Table 1. A total of 19 literatures involved randomized controlled trials (RCTs) on the effects of endurance and strength training sequences on VO_2max_ and lower limb strength performance. The sample size was 482 (male 285 and female 197): 242 in the strength–endurance (S-E) training sequence group and 240 in the endurance–strength (E-S) training sequence group, with an age range of 19–70 years old. Regarding the study samples, 10 and 7 studies included only men and only women, respectively, and two studies included a mixed sampling. A total of 11 studies were on youth research subjects, while 8 were on elderly research subjects. A total of 11 studies involved the VO_2max_ index, among which were the Eklund et al. (2016) and Ruiz-Alias et al. (2022) studies that included male and female subjects; 16 studies were related to lower limb strength performance indicators, which included 21 research works, of which the Eklund et al. (2016) and Ruiz-Alias et al. (2022) studies included male and female subjects, and studies such as those of Lee et al. (2020), Chtara et al. (2008), Shiotsu et al. (2018), and Shiotsu and Yanagita (2018) included two related lower extremity strength test results (Table 2).

**TABLE 1:** Summary of included studies.

Study	Subjects	Sex (M/F)	Strength–endurance sequence (S-E)			Subjects (n)	Endurance–strength sequence (E-S)			Subjects (n)
			Age (y)	Height (cm)	Weight (kg)		Age (y)	Height (cm)	Weight (kg)	
Pinto et al. (2015a)	Elderly	0/21	57.20 ± 2.53	161.57 ± 5.67	66.78 ± 9.0	10	57.09 ± 2.47	158.64 ± 7.64	73.05 ± 13.65	11
Lee et al. (2020)	Youth	20/0	24.5 ± 4.7	179.7 ± 6.5	74.9 ± 10.8	10	24.5 ± 4.7	179.7 ± 6.5	74.9 ± 10.8	10
Pinto et al. (2015b)	Youth	0/26	24.9 ± 2.9	165.4 ± 5.3	64.5 ± 8.1	13	25.4 ± 3.1	162.6 ± 5.6	58.9 ± 5.3	13
Schumann et al. (2014)	Youth	34/0	30 ± 5	179 ± 6	78 ± 11	18	30 ± 5	179 ± 6	78 ± 11	16
Cadore et al. (2013)	Elderly	26/0	64.7 ± 3.7	170.0 ± 5.9	79.7 ± 10.5	13	64.7 ± 4.8	173.5 ± 5.1	83.3 ± 13.4	13
Pinto et al. (2014)	Youth	0/26	24.9 ± 2.9	165.4 ± 5.3	64.5 ± 8.1	13	25.4 ± 3.1	162.6 ± 5.6	58.9 ± 5.3	13
Davitt et al. (2014)	Youth	0/23	19.9 ± 0.4	164.3 ± 2.6	60.4 ± 3.8	10	19.8 ± 0.3	162.9 ± 2.7	61.5 ± 3.4	13
Salamat, (2017)	Youth	26/0	21.66 ± 2.08	174.63 ± 3.48	68.70 ± 3.35	13	22.00 ± 3.00	177.17 ± 4.85	68.72 ± 4.38	13
Esazadeh et al. (2020)	Elderly	0/21	——	——	70 ± 8.4	10	——	——	66.6 ± 9.4	11
Bagheri et al. (2020)	Elderly	20/0	63.8 ± 3.6	166.0 ± 3.8	62.8 ± 2.3	10	61.1 ± 3.3	165.2 ± 4.2	61.1 ± 3.1	10
Costa et al. (2016)	Youth	23/0	16.79 ± 0.93	166.79 ± 9.94	61.65 ± 12.56	12	16.64 ± 0.95	169.64 ± 7.99	61.28 ± 10.36	11
Shiotsu et al. (2018)	Elderly	32/0	69.6 ± 4.6	165.1 ± 6.3	65.7 ± 6.2	16	70.4 ± 4.1	165.6 ± 3.3	64.8 ± 8.4	16
Chtara et al. (2008)	Youth	20/0	21.4 ± 1.3	178.2 ± 5.7	73.69 ± 6.3	10	21.4 ± 1.3	178.2 ± 5.7	75 ± 5.8	10
Mcgawley and Andersson, (2013)	Youth	18/0	23 ± 4	180 ± 8	75.8 ± 6.4	9	23 ± 4	180 ± 8	75.8 ± 6.4	9
Eklund et al. (2016)	Youth	34/29	F 28.9 ± 4.4	F 164.0 ± 5.0	F 62.4 ± 8.0	14 F	F 29.1 ± 5.6	F 168.0 ± 7.0	F 66.7 ± 10.1	15 F
			M 29.8 ± 4.4	M 179.0 ± 5.0	M 75.2 ± 8.5	18 M	M 29.8 ± 6.0	M 178.0 ± 6.0	M 80.3 ± 12.0	16 M
Moghadam et al. (2020)	Elderly	20/0	63.8 ± 3.6	166.0 ± 3.8	62.8 ± 2.3	10	64.1 ± 3.3	165.2 ± 4.2	61.1 ± 3.1	10
Faramarz et al. (2018)	Elderly	0/19	60.34 ± 0.82	155.0 ± 0.1	70.8 ± 3.9	10	60.34 ± 0.82	155.0 ± 0.1	74.66 ± 4.68	9
Shiotsu and Yanagita, (2018)	Elderly	0/24	69.6 ± 4.6	154.1 ± 4.8	51.2 ± 7.4	12	70.4 ± 4.1	153.1 ± 4.5	50.8 ± 6.4	12
Ruiz-Alias et al. (2022)	Youth	12/8	21.0 ± 1.8	179.0 ± 10.0	79.4 ± 11.5	3 F	21.2 ± 2.0	170.0 ± 7.0	63.7 ± 9.9	5 F
						8 M				4 M

M = male; F = female; S-E: strength training first and then endurance training; E-S: endurance training first and then strength training.

**TABLE 2:** Experimental protocols and outcomes of included studies.

Study	Number of weeks	Training frequency per week	Strength training session	Endurance training session	Outcome
Pinto et al. (2015a)	12	2	Bilateral elbow/unilateral hip/bilateral shoulder/unilateral knee flexion, and extension;	3 min of stationary running/3 min of cross-country skiing/3 min of frontal kick, and HR_VT2_ intensity;	Knee extensors
			**1–4 weeks:** 3 sets, duration 20 s, maximal effort intensity 13 min 20 s;	**1–4 weeks:** 2 sets, 18 min;	
			**5–8 weeks:** 4 sets, duration 15 s, maximal effort intensity 16 min 50 s;	**5–8 weeks:** 3 sets, 27 min;	
			**9–12 weeks:** 6 sets, duration 10 s, maximal effort intensity 28 min 20 s	**9–12 weeks:** 4 sets, 36 min	
Lee et al. (2020)	9	3	Gym-based weight training;	Running/cycling/swimming, and various sports (cricket/Australian rules football/soccer/basketball/tennis/volleyball;	Leg press CMJ
			**1–6 weeks:** 3 sets, 6–12 RM;	**1–6 weeks:** 8–12 × 2 min, 85–91% W_peak_;	
			**7–12 weeks:** 4 sets, 6–12 RM	**7–12 weeks:** 8–13 × 2 min, 91–97% W_peak_	
Pinto et al. (2015b)	12	2	Bilateral elbow/unilateral hip/bilateral shoulder/unilateral knee flexion and extension;	3 min of stationary running/3 min of cross-country skiing/3 min of frontal kick, HR_VT2_ intensity;	CMJ VO_2max_
			**1–4 weeks:** 3 sets, duration 20 s, maximal effort intensity 13 min 20 s;	**1–4 weeks:** 2 sets, 18 min;	
			**5–8 weeks:** 4 sets, duration 15 s, maximal effort intensity 16 min 50 s;	**5–8 weeks:** 3 sets, 27 min;	
			**9–12 weeks:** 6 sets, duration 10 s, maximal effort intensity 28 min 20 s	**9–12 weeks:** 4 sets, 36 min	
Schumann et al. (2014)	24	2–3	**1–7/13–18 weeks:** bilateral dynamic leg press;	70 rpm cycling;	Leg press VO_2max_
			**8–12/19–24 weeks:** unilateral dynamic knee extension and flexion and upper body included dynamic seated vertical press and lat pulldown;	**1–7 weeks:** below and above the aerobic threshold (60–70% HR_max_), 30 min;	
			**1–2 weeks:** 2–4 sets, 15–20 repetitions, 40–60% 1RM;	**8–12 weeks:** below and above the anaerobic threshold (80–90% HR_max_), 50 min;	
			**3–10 weeks:** muscle hypertrophy 2–5 sets, 8–10 repetitions, 80–85% 1RM; maximal strength 2–5 sets, 3–5 repetitions, 85–95% 1RM;	**13–24 weeks:** both training volume and intensity were further increased	
			**11–12 weeks:** explosive strength, 8–10 repetitions, 40% 1RM, rest 3–4 min;		
			**13–24 weeks:** strength program structure was maintained, each combined training session was 30–50 min, total duration 60–100 min		
Cadore et al. (2013)	12	3	Bench press/inclined leg press/seated row/knee extension/inverse fly/leg curl/triceps curl/biceps curl abdominal exercises, 40 min;	Cycle ergometer, HR_VT2_ (73.8 ± 4.9% VO_2peak_);	Knee extensors VO_2max_
			**1–2 weeks:** 2 sets, 18–20 RM;	**1–2 weeks:** 20 min, 80% HR_VT_;	
			**3–4 weeks:** 2 sets, 15–17 RM;	**5–6 weeks:** 25 min, 85–90% HR_VT_;	
			**5–7 weeks:** 2 sets, 12–14 RM;	**7–10 weeks:** 30 min, 95% HR_VT_;	
			**8–10 weeks:** 2 sets, 8–10 RM;	**11–12 weeks:** six 4-min bouts 100% HR_VT_	
			**11–12 weeks**: 2 sets, 6–8 RM		
Pinto et al. (2014)	12	2	Bilateral elbow/unilateral hip/bilateral shoulder/unilateral knee flexion and extension;	3 min of stationary running/3 min of cross-country skiing/3 min of frontal kick, HR_VT2_ intensity;	Knee extensors
			**1–4 weeks:** 3 sets, duration 20 s, maximal effort intensity 13 min 20 s;	**1–4 weeks:** 2 sets, 18 min;	
			**5–8 weeks:** 4 sets, duration 15 s, maximal effort intensity 16 min 50 s;	**5–8 weeks:** 3 sets, 27 min;	
			**9–12 weeks:** 6 sets, duration 10 s, maximal effort intensity 28 min 20 s;	**9–12 weeks:** 4 sets, 36 min	
Davitt et al. (2014)	8	4	Three-way split routine (chest and back, shoulders and arms, and lower body);	Aerobic program	Leg press VO_2max_
			**1–8 weeks:** 90–100% 10 RM, 5–6 different exercises, 3 sets, 8–12 repetitions, rest 60–90 s	**1–8 weeks:** 30 min, moderate to moderate–high intensity, 70–80% HRR	
Salamat (2017)	8	3	Bench press/biceps and triceps flexion-extension with weights/underhand cable pull-down/leg press/squat and sit-ups; **1–2 weeks:** 50% 1RM, 25 min **3–6 weeks:** 10% increase every 2 weeks; **7–8 weeks:** 80% 1RM, 45 min	Treadmill; **1–2 weeks:** 55% HR_max_, 25 min; **3–6 weeks:** 10% increase every 2 weeks; **7–8 weeks:** 85% HR_max_, 45 min;	Leg press
					VO_2max_
Esazadeh et al. (2020)	8	3	Biceps curl/triceps pushdown/lat pulldown/lateral raise/incline chest press/leg extension/leg curl/calf raise; **1–8 weeks:** 45 min, rest 10 min	Aerobic exercise (lower and upper body exercises); **Initial sessions:** 65% HR_max_, 20 min; **End of sessions:** 80% HR_max,_ 40 min	Leg extension
Bagheri et al. (2020)	8	3	Leg extension/leg curl/bench press/lat pulldown/lateral raise/abdominal crunch; **1–2 weeks:** 40%–45% 1RM, 2 sets, 14–16 repetitions; **3–4 weeks:** 50%–55% 1RM, 2 sets, 12–14 repetitions; **5–6 weeks:** 60%–65% 1RM, 3 sets, 10–12 repetitions; **7–8 weeks:** 70%–75% 1RM, 3 sets, 8–10 repetitions	Fixed-speed bike; **1–2 weeks:** 55% HR_max_, 15 min; **3–4 weeks:** 60% HR_max_, 20 min; **5–6 weeks:** 65% HR_max_, 25 min; **7–8 week:** 70% HR_max_, 30 min	VO_2max_
Costa et al. (2016)	10	2	Circuit: sit-ups/vertical and horizontal jump/medicine ball throw (1 kg and 3 kg); Medicine ball throw: **1–3 week:** 2 × 8; **4 weeks:** 6 × 8; **5 weeks:** test; **6 weeks:** 4 × 5; **7–8 weeks:** 2 × 5; **9–10 weeks:** 1 × 5; CMJ**:** **1–3 weeks:** 1 × 5; **4 weeks:** 3 × 5; **5 weeks**: test; **6-7 weeks:** 4 × 5; **8 weeks:** 2 × 5; **9–10 week**: 2 × 4;	Shuttle running: 30 × 20 m, MAV: 75%; sprint running: **1–2 weeks:** 4 × 20 m; **3–4 weeks:** 3 × 20 m; **5 weeks:** test; **6–7 weeks:** 4 × 20 m; **8 weeks:** 3 × 40 m; **9–10 weeks:** 2 × 30 m	CMJ VO_2max_
Shiotsu et al. (2018)	10	2	Leg curl/leg press/chest press/seated row/shoulder press; **1–10 weeks:** 3 sets, 70-80% 1RM, 8–12 repetitions, rest 1 min	Cycle ergometer; **1–10 weeks:** 50–55 rpm/min, 60% HRR, 20 min;	Leg press and leg curl
Chtara et al. (2008)	12	2	30 min training, rest 2 min between sets; **1–6 weeks:** strength endurance 1-leg half squats/walking lunges/arm flexion/back extension/hip extension/abdominal; 4 sets, 16–18 repetitions → 5 sets, 20–26 repetitions; **7–12 weeks:** explosive strength training; drop jumps/hurdle jumps/hopping/single-leg hops/single-leg bounds/multiple jumps; Four–five repetitions each movement, the height and distance gradually increase	Running 60% VO_2max_	Peak jump, five jumps
Mcgawley and Andersson (2013)	5	3	Circuit strength training;	Soccer specific fitness training; **Tuesday:** RSA and speed endurance, 5–60 s; **Thursday:** repeated/explosive actions using ladders/hurdles and multi directional running; **Friday:** soccer-specific dribbling track 3V3 SSGs on a 20 × 30 m; 90–95% HR_max_, 4–5 rounds, 4–5 min work, and rest 2–3 min	CMJ
			**Tuesday:** lower body and back core training, 2–3 sets, 5–10 repetitions, 75%–90% 1RM;		
			**Thursday:** lower body and chest core, 2–3 sets, 5–10 repetitions, 75%–90% 1RM;		
			**Friday:** power and core development, 3 sets, 3–20 repetitions		
Eklund et al. (2016)	24	1–12 weeks, four times; 13–24 weeks, five times	Hip extensors/horizontal leg press/seated hamstring curls/seated knee extensions;	Cycle ergometer;	Leg press VO_2max_
			**Initial weeks:** 2–4 sets, 15–20 repetitions, 60% 1RM;	**1–7/13–16 weeks:** anaerobic threshold HR, 30–50 min;	
			**Hypertrophy period:** 2–5 sets, 8–12 repetitions, 80%–85% 1RM;	**8–12/17–24 weeks:** anaerobic threshold HR, 10–15 min, anaerobic threshold∼ aerobic threshold HR, 5 min	
			**Maximal strength period:** 2–5 sets, 3–5 repetitions, 85%–95% 1RM	X	
Moghadam et al. (2020)	8	3	Leg extension/leg curl/bench press/lat pulldown/lateral raise/abdominal crunch;	Cycling on a fixed-speed cycle ergometer;	VO_2max_
			**1 week:** 40% 1RM, 2 sets, 16–18 repetitions;	**1 week:** 55% HR_max_, 15 min;	
			**8 weeks:** 75% 1RM, 3 sets, 8–10 repetitions	**8 weeks:** 70% HR_max_, 30 min	
Faramarz et al. (2018)	8	3	Bench press/leg press/bent over lateral pull down/bilateral biceps curl/bilateral triceps pushdown;	Cycle ergometer;	Leg curl VO_2max_
			**1–2 weeks:** 2 sets, 40–45% 1RM, 16–18 repetitions;	**1–2 weeks:** 60–66% HR_max_, 16 min;	
			**3–4 weeks:** 2 sets, 50–55% 1RM, 12–14 repetitions;	**3–4 weeks:** 70–74% HR_max_, 20 min;	
			**5–6 weeks:** 3 sets, 60–65% 1RM, 10–12 repetitions;	**5–6 weeks:** 77–80% HR_max_, 25 min;	
			**7–8 weeks:** 3 sets, 70–75% 1RM, 8–10 repetitions	**7–8 weeks:** 85–88% HR_max_, 30 min	
Shiotsu and Yanagita (2018)	10	2	Leg curl/leg press/chest press/seated row/shoulder press;	Cycle ergometer; **1–10 weeks:** 50–55 rpm/min, 60% HRR, 20 min	Leg press and leg curl
			**1–10 weeks:** 3 sets, 70–80% 1RM, 8–12 repetitions, rest 1 min		
Ruiz-Alias et al. (2022)	8	3	Bench press, back squat	Running, SIT	CMJ VO_2max_
			**1–2 weeks:** 4–5 × 60% 1RM, 5–6 repetitions, rest 2 min;	**1–2 weeks:** 4 × 30 s all out, 4 min active recovery	
			**3–4 weeks:** 5–6 × 70% 1RM, 3–4 repetitions, rest 2 min;	**3–4 weeks:** 5 × 30 s all out, 4 min active recovery	
			**5–6 weeks:** 5–6 × 80% 1RM, 2–3 repetitions, rest 2 min;	**5–6 weeks:** 6 × 30 s all out, 4 min active recovery	
			**7–8 weeks:** 6 × 60% 1RM, 1–2 repetitions, rest 2 min	**7–8 weeks:** 6 × 30 s all out, 4 min active recovery	

HR, heart rate; HR_max_, heart rate max; HR_VT2_, heart rate corresponding to the second ventilatory threshold; HRR, HR reserve; MAV, maximum individual aerobic velocity; CMJ, counter movement jump; RSA, repeated sprint ability; SSG, small side games; SIT, sprint interval training.

In the 19 included studies, the outcome measures for evaluating lower limb strength performance included leg press, leg curl, knee extensors, CMJ, and five jumps. The exercise interventions consisted of simultaneous strength and endurance training, but with different training sequences, in which the training period ranged from 8 to 24 weeks and the training was performed 2–3 times a week. Strength training was mainly based on circuit training, and the training intensity increased with the number of training weeks. Endurance training was mainly based on power bikes and aerobic running. Individual studies included kicking, skiing, and other movements. The specific training plan is shown in Table 2.

### 3.2 Risk of bias assessment

**FIGURE 2:**
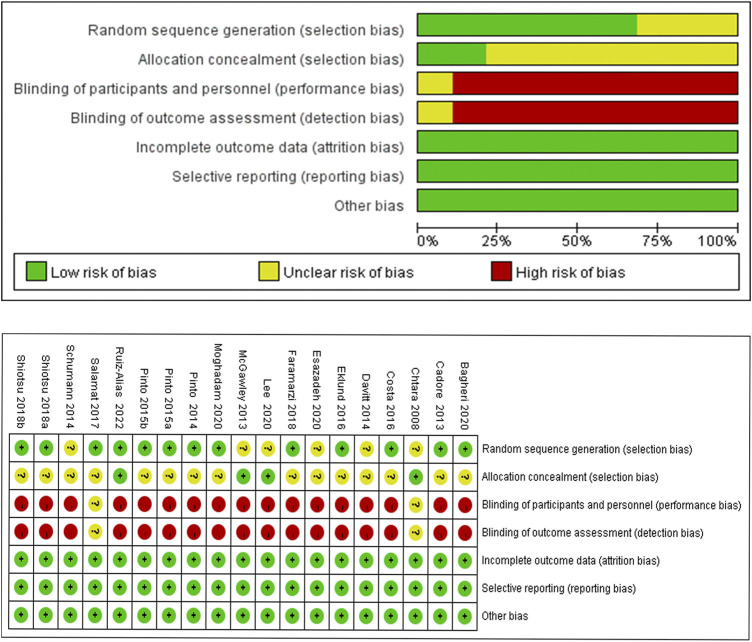
Summary of risk of bias of the studies included.

The 19 studies included in this article were all randomized controlled trials, and the quality assessment is shown in Figure 2. Among them, 68.4% (13/19) clearly reported random sequence generation and 21.1% (4/19) reported allocation concealment; insufficient performance of research blinding, not concealing experimental purpose, and training or testing content from subjects when signing the informed consent form resulted in a high risk of implementation bias. The reasons may be related to factors such as the nature of the experiment, the experimental environment, and the particularity of the subjects themselves; the quality of the report on the outcome data, selective reporting, and other bias were all reported completely.

### 3.3 Effects of interventions

#### 3.3.1 Effects of concurrent training sequence on VO_2max_

Data on VO_2max_ were obtained from 11 studies comprising a total of 300 individuals. In the Eklund et al. (2016) study, males and females were subjected to simultaneous training interventions, so the meta-analysis finally included 12 effect size data or percentage change data of VO_2max_ change before and after the interventions.

**FIGURE 3:**
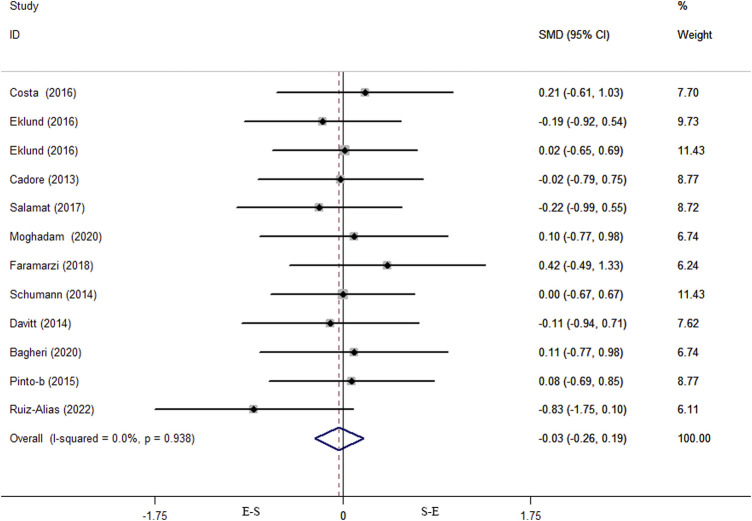
Forest plot of effects of concurrent training sequence on VO_2max_.

**FIGURE 4:**
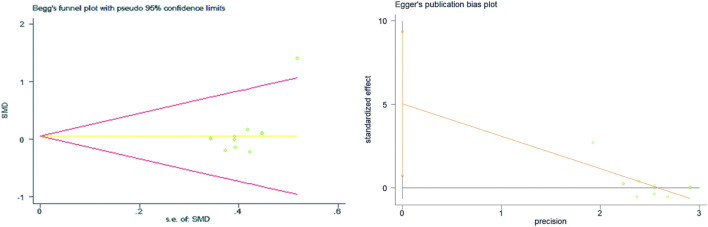
Bias analysis of the impact of concurrent training sequence on VO_2max_.

The results of the heterogeneity test showed that there was no heterogeneity among the studies (*p* = 0.578, I^2^ = 0%), so the fixed effects model was selected for analysis. No difference was found between the S-E and E-S training sequence interventions on VO_2max_ (SMD = 0.02, 95% CI: −0.21–0.25, *p* = 0.859) (Figure 3). The results of the Begg’s test (Z = 0.89, *p* = 0.373) and Egger’s test (T = 1.6, *p* = 0.133) both indicated that there was no publication bias (Figure 4). After the trim-and-fill test, it was found that there was no study of trimming and supplementation, and the data did not change. Sensitivity analysis was not performed for VO_2max_ results because there was no heterogeneity.

In order to further explore the possible influencing factors of S-E and E-S training sequences on VO_2max_, the results of 12 VO_2max_ research studies were analyzed in subgroups based on age, gender, training period, and training frequency. Subgroup analysis found that no matter the E-S training or S-E sequence, there was no difference in VO_2max_ under different age, gender, training period, and training frequency (*p* > 0.05), indicating that the order of endurance and strength training had no significant effect on VO_2max_ (Table 3).

**TABLE 3:** Subgroup analysis of the impact of concurrent training sequence on VO_2max_.

Factors	Subgroups	Studies	Subjects		SMD (95% CI)	I^2^	Z	Weight%
			S-E	E-S				
Age	Youth	Eklund et al. (2016), Pinto et al. (2015b), Schumann et al. (2014), Davitt et al. (2014), Salamat (2017), Costa et al. (2016), Ruiz-Alias et al. (2022)	109	106	−0.09	I^2^ = 0% (*P* = 0.976)	Z = 0.64 (*P* = 0525)	72.49%
					(−0.36,0.18)			
	Old	Cadore et al. (2013), Bagheri et al. (2020), Moghadam et al. (2020), Faramarz et al. (2018)	43	42	0.305	I^2^ = 46.0% (*P* = 0.135)	Z = 1.37 (*P* = 0.171)	27.51%
					(−0.13,0.74)			
Gender	Man	Eklund et al. (2016), Schumann et al. (2014), Cadore et al. (2013), Salamat (2017), Bagheri et al. (2020), Costa et al. (2016), Moghadam et al. (2020)	94	89	0.02 (−0.27,0.31)	I^2^ = 0% (*P* = 0.999)	Z = 0.16 (*P* = 0.873)	66.46%
	Woman	Eklund et al. (2016), Faramarz et al. (2018), Pinto et al. (2015b), Davitt et al. (2014)	47	50	0.12	I^2^ = 0% (*P* = 0.619)	Z = 0.59 (*P* = 0.556)	33.54%
					(−0.29,0.53)			
Training period	>8	Eklund et al. (2016), Pinto et al. (2015b), Schumann et al. (2014), Cadore et al. (2013), Costa et al. (2016)	88	84	0.00	I^2^ = 0% (*P* = 0.993)	Z = 0.02 (*P* = 0.983)	58.36%
					(−0.30,0.30)			
	≤8	Davitt et al. (2014), Salamat (2017), Bagheri et al. (2020), Moghadam et al. (2020), Faramarz et al. (2018), Ruiz-Alias et al. (2022)	64	64	0.06	I^2^ = 44.1% (*P* = 0.111)	Z = 0.30 (*P* = 0.763)	41.64%
					(−0.30,0.41)			
Training frequency	>2	Eklund et al. (2016). Schumann et al. (2014), Cadore et al. (2013), Davitt et al. (2014), Bagheri et al. (2020), Salamat (2017), Moghadam et al. (2020), Faramarz et al. (2018), Ruiz-Alias et al. (2022)	127	124	0.004	I^2^ = 3.6% (*P* = 0.407)	Z = 0.03 (*P* = 0.974)	83.37%
					(−0.25,0.26)			
	=2	Pinto et al. (2015b), Costa et al. (2016)	25	24	0.10	I^2^ = 0% (*P* = 0.839)	Z = 0.36 (*P* = 0.716)	16.63%
					(−0.46,0.67)			

#### 3.3.2 Effects of concurrent training sequence on lower limb strength performance

**FIGURE 5:**
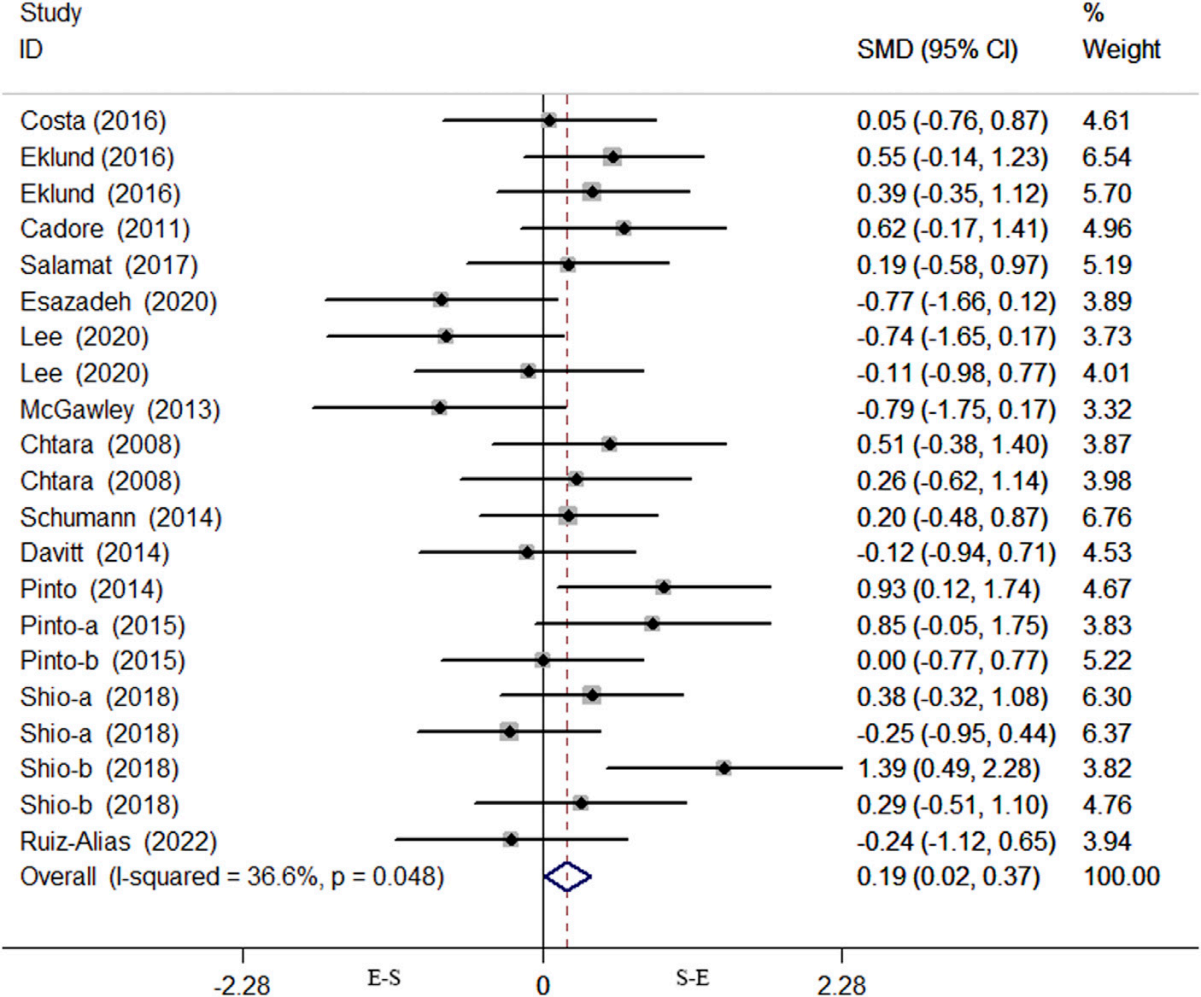
Forest plot of effects of concurrent training sequence on lower limb strength.

**FIGURE 6:**
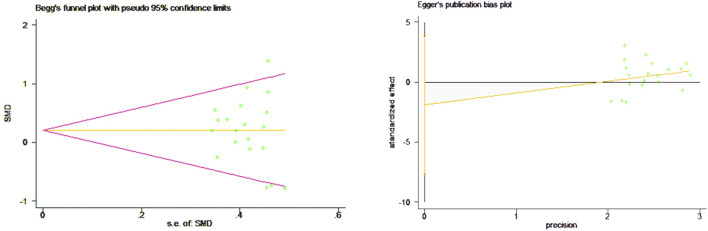
Bias analysis of the impact of concurrent training sequence on lower limb strength.

Data on VO_2max_ were obtained from 16 studies comprising a total of 519 individuals, and the meta-analysis included 21 effect size data or percentage change data of lower limb strength performance change before and after the intervention. The results of the heterogeneity test showed that there was heterogeneity among the studies (*p* = 0.048, I^2^ = 36.6%), and a fixed effects model was selected for analysis. As shown in Figure 5, there was a significant difference in lower limb strength performance before and after the S-E and E-S sequence interventions (SMD = 0.19, 95% CI: 0.02–0.37, *p* = 0.032), and the S-E sequence showed an advantage in favor of lower limb strength performance. The results of the Begg’s test (Z = 0.82, *p* = 0.415) and Egger’s test (T = −0.85, *p* = 0.404) both indicated that there was no publication bias (Figure 6).

**FIGURE 7:**
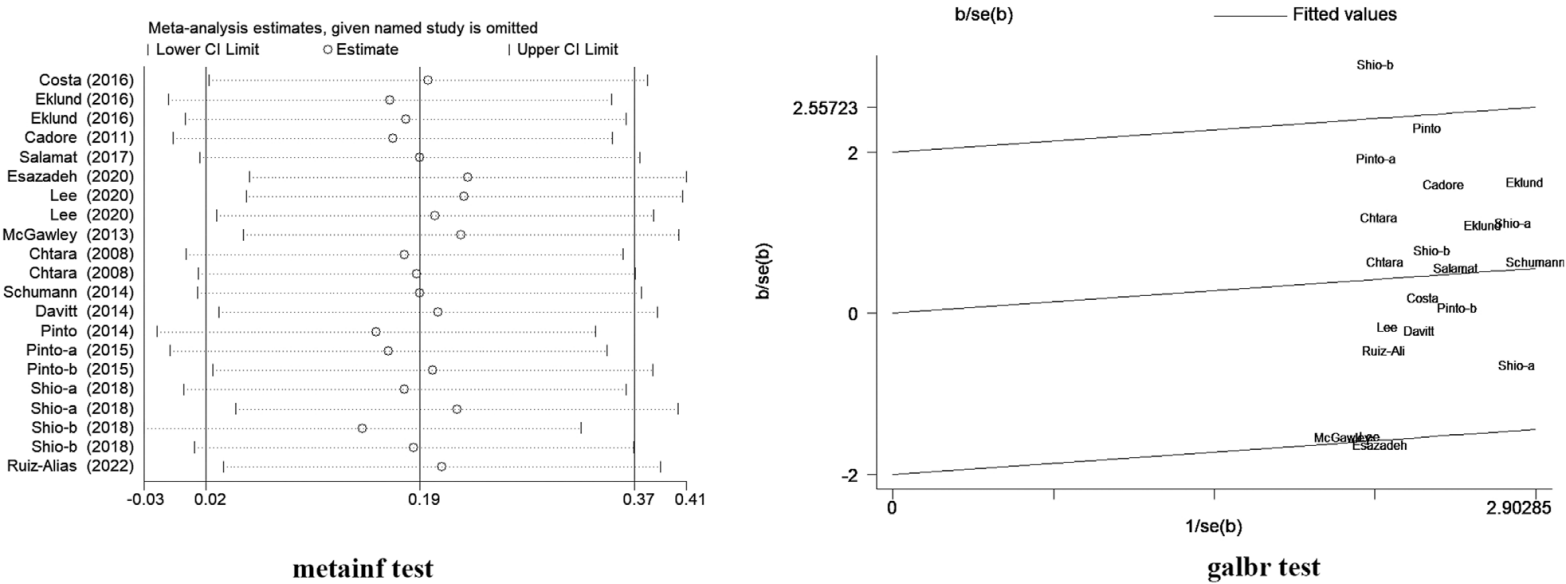
Sensitivity analysis of concurrent training sequence on lower limb strength.

The heterogeneity of the lower extremity strength index in the 16 studies was 36.6%. Owing to the heterogeneity found between the studies, sensitivity analyses were performed by metainf and galbr tests (Figure 7), and it was found that the Shiotsu and Yanagita (2018) leg curl test and Esazadeh et al. (2020) leg extension test may be the reason for the slightly higher heterogeneity. However, the training programs of the two studies have no special differences in comparison with other studies, and the sensitivity of the results of another study by Shiotsu and Yanagita (2018) met the standard of leg press. Excluding these two studies, the heterogeneity was reduced to 5%; there was no significant change in the results of the meta-analysis, indicating that the results of the meta-analysis in this study were more reliable; and the study was retained.

The changes of S-E and E-S sequences on lower limb strength performance may be affected by other interference factors; the results of 21 research studies were analyzed in subgroups based on age, gender, training period, training frequency, and strength performance (Table 4). We found that the S-E sequence of old people showed an advantage in favor of lower limb strength performance increase than of youth (*p* = 0.039), the S-E sequence of females was more conducive to the growth of lower limb strength than of males (*p* = 0.017). The S-E sequence of more than 8 weeks showed greater advantage in improving the strength of the lower limb than of the E-S training sequence (*p* = 0.002); the training within 8 weeks to improve the strength of the lower limb was biased toward the E-S sequence (*p* = 0.032). The twice a week of S-E training sequence was more advantageous to the growth of lower body strength (*p* = 0.003). The E-S training method is preferred to improve the jumping power of the lower limbs, and the S-E sequence showed the advantage of improving the maximum strength of the lower limb knee extension (*p* = 0.026) and leg curl (*p* = 0.004).

**TABLE 4:** Subgroup analysis of the impact of concurrent training sequence on lower limb strength.

Factors		Subgroups		Studies	Subjects		SMD (95% CI)	I^2^	Z	Weight%
					S-E	E-S				
Age		Youth		Eklund et al. (2016), Lee et al. (2020), Pinto et al. (2015b), Schumann et al. (2014), Pinto et al. (2014), Davitt et al. (2014), Salamat (2017), Costa et al. (2016), Mcgawley and Andersson (2013), Faramarz et al. (2018), Ruiz-Alias et al. (2022)	171	168	0.13	I^2^ = 12.6% (*P* = 0.315)	Z = 1.16 (*P* = 0.247)	66.08%
							(−0.09,0.34)			
		Old		Shiotsu et al. (2018), Shiotsu and Yanagita (2018), Pinto et al. (2015a), Cadore et al. (2013), Esazadeh et al. (2020)	89	91	0.32	I^2^ = 61.7% (*P* = 0.016)	Z = 2.06 (*P* = 0.039)	33.92%
							(0.02,0.62)			
Gender		Man		Chtara et al. (2008), Eklund et al. (2016), Lee et al. (2020), Shiotsu et al. (2018), Schumann et al. (2014), Salamat (2017), Costa et al. (2016), Mcgawley and Andersson (2013)	155	150	0.12	I^2^ = 13.4% (*P* = 0.314)	Z = 1.04 (*P* = 0.298)	62.1%
							(−0.11,0.35)			
		Woman		Eklund et al. (2016), Shiotsu and Yanagita (2018), Pinto et al. (2015a), Pinto et al. (2015b), Pinto et al. (2014), Davitt et al. (2014), Esazadeh et al. (2020)	94	100	0.36	I^2^ = 57.3%(*P* = 0.022)	Z = 2.39(*P* = 0.017)	37.9%
							(0.06,0.65)			
Training period		>8		Eklund et al. (2016), Lee et al. (2020), Shiotsu et al. (2018), Shiotsu and Yanagita (2018), Pinto et al. (2015a), Pinto et al. (2015b), Schumann et al. (2014), Cadore et al. (2013), Costa et al. (2016)	207	204	0.32	I^2^ = 25.1% (*P* = 0.171)	Z = 3.17 (*P* = 0.002)	79.13%
							(0.12,0.52)			
		≤8		Davitt et al. (2014))^,^ Salamat (2017), Esazadeh et al. (2020), Mcgawley and Andersson (2013), Ruiz-Alias et al. (2022)	53	55	−0.29	I^2^ = 0% (*P* = 0.426)	Z = 1.49 (*P* = 0.032)	20.87%
							(−0.68,0.09)			
Training frequency		>2		Eklund et al. (2016), Lee et al. (2020), Schumann et al. (2014), Cadore et al. (2013), Davitt et al. (2014), Salamat (2017), Esazadeh et al. (2020), Mcgawley and Andersson (2013), Ruiz-Alias et al. (2022)	136	135	0.02	I^2^ = 32.6% (*P* = 0.138)	Z = 0.14 (*P* = 0.890)	52.58%
							(−0.23,0.26)			
		=2		Chtara et al. (2008), Shiotsu et al. (2018), Shiotsu and Yanagita (2018), Pinto et al. (2015a), Pinto et al. (2015b), Pinto et al. (2014), Costa et al. (2016)	124	124	0.39	I^2^ = 28.1% (*P* = 0.186)	Z = 2.96 (*P* = 0.003)	47.42%
							(0.13,0.64)			
Sport performance		Knee extension strength		Pinto et al. (2015a), Cadore et al. (2013), Pinto et al. (2014), Esazadeh et al. (2020)	46	48	0.44	I^2^ = 68.4% (*P* = 0.023)	Z = 2.05 (*P* = 0.040)	17.35%
							(0.02,0.86)			
		Leg curl strength		Eklund et al. (2016), Lee et al. (2020), Shiotsu et al. (2018), Shiotsu and Yanagita (2018), Schumann et al. (2014), Davitt et al. (2014), Salamat (2017)	139	139	0.27	I^2^ = 14.6% (*P* = 0.309)	Z = 2.22 (*P* = 0.026)	53.98%
							(0.03,0.51)			
		Power		Chtara et al. (2008), Lee et al. (2020), Pinto et al. (2015b), Costa et al. (2016), Mcgawley and Andersson (2013), Ruiz-Alias et al. (2022)	75	72	−0.11	I^2^ = 9.1% (*P* = 0.359)	Z = 0.64 (*P* = 0.523)	28.67%
							(−0.44,0.22)			

## 4 Discussion

This systematic review quantified the effects of S-E and E-S concurrent training sequences on aerobic and lower limb strength abilities. We compared the effects of two training modes on VO_2max_ and knee extension strength, leg curl strength, and jump power. The E-S sequence will not effect changes in VO_2max_; the S-E sequence may be more conducive in improving lower limb strength. In addition, we further addressed the subgroups in the discussion according to age, sex, training period, training frequency, and motor performance.

### 4.1 Effect of concurrent training sequence on aerobic capacity

#### 4.1.1 Effect of concurrent training sequence on VO_2max_ and sport performance

VO_2max_ is a classic indicator to measure the level of cardiopulmonary function and aerobic endurance. Common endurance training includes continuous training, high-intensity interval training, and lactate threshold intensity training, which often consist of longer endurance training sessions. At present, it is believed that long-term endurance training affects the growth of strength ability, while certain strength training has no obvious effect on endurance ability, and the aerobic and strength concurrent training on endurance ability is less than that of strength ability (Wilson et al., 2012). However, there is still no systematic report on whether the sequence of aerobic and strength training affects endurance ability. In the 11 data of S-E and E-S groups before and after the experiments that were included in this study, VO_2max_ was found to show an increasing trend in the two groups, but there was no significant difference between the two groups. The results of the meta-analysis also proved that the effect of the concurrent training sequence on VO_2max_ was not significant. Statistical significance (SMD = 0.02, 95% CI: −0.21–0.25, *p* > 0.05), low heterogeneity, and publication bias further improve the reliability of this evidence.

Usually, the increase of VO_2max_ is often accompanied by a richer capillary network, an increase in the number of mitochondria, and an increase in oxidase activity. This may be related to the level of pulmonary ventilation and gas exchange in the lungs, the ability of the blood and circulatory system to transport oxygen, and the increased ability of muscle tissue to utilize oxygen. There was no difference in the effects of endurance and strength training sequence on the aforementioned influencing factors. In addition, some studies have found in male and female athletes that concurrent training could improve the peak running speed and running economy of the athletes (Barnes et al., 2013). Strength training after 12 weeks of HIIT can better improve 4 km running performance and maximal aerobic speeds (vVO_2max_) than the reverse training sequence (Chtara et al., 2005), which may be because strength training will first lead to skeletal muscle soreness, nerve fatigue, reduced adaptation to endurance ability, and reduced exercise economy (Doma et al., 2017), resulting in a decrease in the percentage utilization level of VO_2max_ in the aerobic energy supply phase, reducing sport performance.

#### 4.1.2 Effect of concurrent training sequence on VO_2max_ subgroups

In addition to respiratory, blood, cardiovascular, and skeletal muscles, the influencing factors of VO_2max_ are also affected by other factors such as age, gender, and training. Previous studies have shown that concurrent training could improve the cardiorespiratory level of the elderly better than simple aerobic training and would not affect the aerobic adaptation produced by endurance training alone (SillanPää et al., 2009; Karavirta et al., 2011). Ferrari et al. (2013) found that the increase of VO_2peak_ in the elderly would not seem to be affected by training frequency, and twice a week of concurrent training may be the optimal training frequency for the elderly, which can maximize the increase in muscle strength and cardiorespiratory function. For the concurrent training sequence, our study found no differences between S-E and E-S training sequences for training twice a week (SMD = 0.10, 95% CI: −0.46–0.66, *p* = 0.716) or more than twice a week (SMD = 0.004, 95% CI: −0.25–0.26, *p* = 0.974) by subgroup analysis of training frequency. Therefore, when the elderly improve their cardiorespiratory endurance, it is better to perform endurance and strength training suitable for individuals twice a week, according to their personal conditions. A study on males and females (Schumann et al., 2015) found that after one or more days of strength and endurance training, physiological changes in males and females were similar under different training methods. The subgroup analyses of the concurrent training sequence by gender yielded consistent results in this study (Table 3).

At present, in the research on the concurrent training sequence, the aerobic training program mostly adopts the bicycle riding mode. In this study, only Cadore et al. (2013) conducted aerobic running on a treadmill. Wilson et al. (2012) also pointed out in their meta-analysis that cycling training can reduce the incompatible effect of endurance on strength. This may be because running plays a significant role in training practice, and the muscles experience a more eccentric contraction process and stress stimulation to the body (Doma et al., 2019) and this exerts considerable load on the musculoskeletal system. To avoid excessive loading, low-impact alternatives are often introduced in training practice, such as cycling, rowing ergometer training, or water aerobics training; therefore, we speculate that the aerobic training methods of running or cycling may have different effects on VO_2max_ in different concurrent training sequences.

### 4.2 Effect of concurrent training sequence on performance of lower body strength

This part of the discussion mainly analyzes the effects of concurrent endurance and strength training sequence on lower limb muscle strength in order to find a more suitable training program. This study has found that the S-E training sequence improved lower body strength better than the E-S training sequence (SMD = 0.19, 95% CI: 0.02–0.37, *p* = 0.523), which may be related to the inhibition of muscle fiber hypertrophy caused by muscle glycogen depletion that is caused by endurance training (Mcbride et al., 2009). Its molecular mechanism is that endurance training activates adenosine monophosphate–activated kinase (AMPK), strengthens mitochondrial function, promotes improvement of the endurance level, and inhibits mammalian target of rapamycin (mTOR) activation and affects muscle protein synthesis.

Strength training can cause mechanical disorders of muscle cells, promote the secretion of insulin-like growth factors-1 (IGF-1), and subsequently, upregulate the phosphorylation of mTOR to activate its function (Bodine, 2006). In addition, strength training stimulates testosterone increases more than aerobic training does, which may be related to the fact that the anaerobic glycolytic pathway severely affects testosterone increase (Kraemer and Ratamess, 2005); however, the S-E sequence may attenuate this effect.

#### 4.2.1 Effect of training period and training frequency on strength in different concurrent training sequences

Usually, strength training can stimulate muscle strength growth for 4–8 weeks, while distance runners do not observe strength gains after mixed strength training (MIX) (Beattie et al., 2017), it can be seen that endurance has a certain interference effect on the improvement of strength. The results of the subgroup analysis showed that when concurrent training was for more than 8 weeks, the S-E sequence (SMD = 0.32, 95% CI: 0.12–0.52, *p* = 0.002) showed more advantages to improve lower limb strength. Muscle strength may be better improved by the E-S sequence (SMD = −0.29, 95% CI: 0.68–0.09, *p* = 0.032) when training for less than 8 weeks. Therefore, the S-E training sequence can be used when improving lower body strength and a training period of more than 8 weeks can avoid the interference of endurance on strength ability.

Concurrent training frequency was shown to affect adaptive responses, with improvements in muscle strength diminishing when strength and endurance training was performed 4–6 times per week (Kraemer et al., 1995). A meta-analysis of 21 articles of 422 people on the effects of concurrent training on strength showed that endurance training no more than thrice a week can effectively reduce the incompatibility of training (Wilson et al., 2012). Similar results were found in this study, when training twice a week, and S-E training showed more advantages in improving lower body strength (SMD = 0.39, 95% CI: 0.13–0.64, *p* = 0.03), suggesting that it is better to use the low frequency method to perform the S-E sequence during concurrent training.

#### 4.2.2 Effect of age and gender on strength in different training sequences

It has been reported by Cadore et al. (2012, 2013) that the S-E sequence in the elderly shows a better maximal dynamic strength improvement effect and a greater relative muscle strength improvement than the E-S sequence. The results of this study are similar, in that both the elderly (SMD = 0.15, 95% CI: 0.32–0.62, *p* = 0.039) and young people (SMD = 0.15, 95% CI: 0.07–0.37, *p* = 0.185) tend to improve their lower extremity strength in the S-E sequence. Cadore et al. (2012) found that older men who performed aerobic exercise after 12 weeks of resistance training were more effective in increasing strength, while Shiotsu et al. (2018) found that with 10 weeks, 2–3 times a week, of concurrent training, the lower limb strength of the elderly increased but was not affected by the order of endurance and strength training, which is consistent with our recommendation that concurrent training is best for more than 8 weeks.

Referring to the Rønnestad et al. (2010, 2011) strength training program, Vikmoen et al. (2016a, 2017) compared gender differences in female cyclists and found that male and female cyclists had similar effects on all aspects of muscle strength, muscle hypertrophy, and cycling ability after 12 weeks of heavy strength training. In addition, the lactate threshold and performance in the Wingate test showed similar increases in the 40-min full-strength test, but there was no significant difference, indicating that adding strength training to normal training had no gender difference in the improvement of cycling performance. Similarly, female athletes with a good endurance training background were trained for 11 weeks of concurrent training (Vikmoen et al., 2016b), and it was found that compared with strength training alone, women who trained during concurrent training showed smaller improvements in strength, proving that female athletes also have interference effects. In this study, we found from the gender subgroup analysis that the S-E sequence of females (SMD = 0.36, 95% CI: 0.06, 0.65, *p* = 0.017) was more effective than that of males (SMD = 0.12, 95% CI: 0.11–0.35, *p* = 0.298 > 0.05) on the improvement of lower limb strength, indicating that the concurrent training sequence may be one of the interference effects of male and female strength. This may be related to the difference in serum testosterone hormone levels in men and women (Nindl et al., 2016), in which women use more fat for energy use, while men have a higher ratio of protein and carbohydrates.

#### 4.2.3 Effect of lower body strength performance in different training sequences

The results of the subgroup analysis of lower power performance in this study were SMD = −0.11, 95% CI: 0.44–0.22, and *p* = 0.632, that is, the order of endurance and strength training had little effect. Balabinis et al. (2003) verified that high-intensity short-duration sprint training for basketball players did not affect maximum strength and power strength; Yu (2014) proposed that strength training no more than thrice a week is the optimal load frequency stimulation to improve muscle strength and power of athletes. The development of 8–10RM of local muscle endurance (LME) and aerobic work at training intensity can effectively reduce incompatibility. It has been suggested that the results of the studies included in our article are not of simple aerobic power cycling or running mode in aerobic training but of sprint running, variable speed running, kicking, skiing, and other movements, and that strength training pays more attention to power and high-intensity training.

In elite kayakers, García-pallarés et al. (2009) found that the S-E training sequence, or a 6–8 h interval between endurance and strength training, ensured restoration of muscle glycogen stores and improved aerobic work, maximal strength, and power. Our study confirmed that the S-E sequence was beneficial to the improvement of maximal flexion and extension of the lower limbs for maximal muscle strength in knee extension (SMD = 0.44, 95% CI: 0.02–0.86, *p* = 0.040) and leg curl (SMD = 0.27, 95% CI: 0.03–0.51, *p* = 0.026). However, there is still insufficient evidence to compare the effects of the S-E training sequence on the improvement of knee flexion and extension strength, which may be related to specific strength training positions. This study involved only four studies on knee extension strength. In the future, the concurrent training sequence will also be considered to analyze the different indicators of lower limb strength evaluation, one by one.

## 5 Strength and limitations

Many sports require both endurance and strength abilities. However, in the training process, incompatibility of endurance and strength is a problem that cannot be ignored. It is very important for us to find a reasonable training sequence and avoid physical adaptation caused by endurance training affecting the development of maximum muscle strength and power. This review was conducted with a meta-analysis to examine the effects of the concurrent training sequence on VO_2max_ and lower limb performance. The available data can provide us with a reasonable sequence of endurance and strength training, to improve VO_2max_ and lower limb strength performance. Furthermore, our data provide some preliminary insights into the cycle and frequency of endurance and strength training for the elderly and women.

Due to different characteristics of different sports, the proportion of endurance and strength training is different. The available data from the literature concerning underlying we were not obtained the respective proportions of endurance and strength training load during the concurrent training. Therefore, it has been temporarily impossible to analyze the abilities that are dominant and the sequence that is advantageous in endurance and strength training in different sports. In addition, we could not obtain enough data to further classify adolescents and children in the youth subgroup. As we know the sensitive periods of physical fitness development for children, it is important for children to discuss the proportions of general athletic development training to specific skill training and know the load of endurance training and the way of strength training. We could not clarify whether interference effects in strength adaptations are more pronounced in adolescents than in children.

## 6 Conclusion

The findings of this systematic review and meta-analysis could provide helpful guidance on exercise prescription: concurrent endurance and strength training sequence will not affect the change of VO_2max_; strength training first and then endurance training may be more conducive when improving the strength of knee flexion and knee extension. In the elderly and female population, a training period of more than 8 weeks and the training frequency of twice a week are more advantageous for the improvement of lower body strength in strength training first and then in endurance training sequence.

## Data Availability

The original contributions presented in the study are included in the article/Supplementary Material; further inquiries can be directed to the corresponding author.

## References

[B1] Bagheri R. Moghadam B. H. Church D. D. Tinsley G. M. Eskandari M. Moghadam B. H. (2020). The effects of concurrent training order on body composition and serum concentrations of follistatin, myostatin and GDF11 in sarcopenic elderly men. Exp. Gerontol. 133, 110869. 10.1016/j.exger.2020.110869 32035222

[B2] Balabinis C. P. Psarakis C. H. Moukas M. Vassiliou M. P. Behrakis P. K. (2003). Early phase changes by concurrent endurance and strength training. J. Strength Cond. Res. 17 (2), 393–401. 10.1519/1533-4287(2003)017<0393:epcbce>2.0.co;2 12741884

[B3] Barnes K. R. Hopkins W. G. Mcguigan M. R. Northuis M. E. Kilding A. E. (2013). Effects of resistance training on running economy and cross-country performance. Med. Sci. Sports Exerc 45 (12), 2322–2331. 10.1249/MSS.0b013e31829af603 23698241

[B4] Beattie K. Carson B. P. Lyons M. Rossiter A. Kenny I. C. (2017). The effect of strength training on performance indicators in distance runners. J. Strength Cond. Res. 31 (1), 9–23. 10.1519/JSC.0000000000001464 27135468

[B5] Bodine S. C. (2006). mTOR signaling and the molecular adaptation to resistance exercise. Med. Sci. Sports Exerc 38 (11), 1950–1957. 10.1249/01.mss.0000233797.24035.35 17095929

[B6] Cadore E. L. Izquierdo M. Alberton C. L. Pinto R. S. Conceicao M. Cunha G. (2012). Strength prior to endurance intra-session exercise sequence optimizes neuromuscular and cardiovascular gains in elderly men. Exp. Gerontol. 47 (2), 164–169. 10.1016/j.exger.2011.11.013 22178632

[B7] Cadore E. L. Izquierdo M. Pnto S. S. Alberton C. L. Pinto R. S. Baroni B. M. (2013). Neuromuscular adaptations to concurrent training in the elderly: Effects of intrasession exercise sequence. Age (Dordr) 35 (3), 891–903. 10.1007/s11357-012-9405-y 22453934 PMC3636398

[B8] Castro-piñero J. Ortega F. B. Artero E. G. Girela-Rejon M. J. Mora J. Sjostrom M. (2010). Assessing muscular strength in youth: Usefulness of standing long jump as a general index of muscular fitness. J. Strength Cond. Res. 24 (7), 1810–1817. 10.1519/JSC.0b013e3181ddb03d 20555277

[B9] Chtara M. Chamari K. Chaouachi M. ChAouAchi A. Koubaa D. Feki Y. (2005). Effects of intra-session concurrent endurance and strength training sequence on aerobic performance and capacity. Br. J. Sports Med. 39 (8), 555–560. 10.1136/bjsm.2004.015248 16046343 PMC1725284

[B10] Chtara M. Chaouachi A. Levin G. T. Chaouachi M. Chamari K. Amri M. (2008). Effect of concurrent endurance and circuit resistance training sequence on muscular strength and power development. J. Strength Cond. Res. 22 (4), 1037–1045. 10.1519/JSC.0b013e31816a4419 18545210

[B11] Costa A. M. Gil M. H. Sousa A. C. Ensinas V. Pereira A. (2016). Effects of concurrent strength and endurance training sequence order on physical fitness performance in adolescent students. JPE 16 (4), 1202–1206.

[B12] Davitt P. M. Pellegrino J. K. Schanzer J. R. Tjionas H. Arent S. M. (2014). The effects of a combined resistance training and endurance exercise program in inactive college female subjects: Does order matter? J. Strength Cond. Res. 28 (7), 1937–1945. 10.1519/JSC.0000000000000355 24378658

[B13] Docherty D. Sporer B. (2000). A proposed model for examining the interference phenomenon between concurrent aerobic and strength training. Sports Med. 30 (6), 385–394. 10.2165/00007256-200030060-00001 11132121

[B14] Doma K. Deakin G. B. Bentley D. J. (2017). Implications of impaired endurance performance following single bouts of resistance training: An alternate concurrent training perspective. Sports Med. 47 (11), 2187–2200. 10.1007/s40279-017-0758-3 28702901

[B15] Doma K. Deakin G. B. Schumann M. Bentley D. J. (2019). Training considerations for optimising endurance development: An alternate concurrent training perspective. Sports Med. 49 (5), 669–682. 10.1007/s40279-019-01072-2 30847824

[B16] Doma K. Deakin G. B. (2013). The effects of strength training and endurance training order on running economy and performance. Appl. Physiol. Nutr. Metab. 38 (6), 651–656. 10.1139/apnm-2012-0362 23724883

[B17] Eklund D. Häkkinen A. Laukkanen J. A. Balandzic M. Nyman K. Hakkinen K. (2016). Fitness, body composition and blood lipids following 3 concurrent strength and endurance training modes. Appl. Physiol. Nutr. Metab. 41 (7), 767–774. 10.1139/apnm-2015-0621 27351384

[B18] Eng C. Kramer C. K. Zinman B. Retnakaran R. (2014). Glucagon-like peptide-1 receptor agonist and basal insulin combination treatment for the management of type 2 diabetes: A systematic review and meta-analysis. Lance 384 (9961), 2228–2234. 10.1016/S0140-6736(14)61335-0 25220191

[B19] Esazadeh L. Khajeie R. Hosseinikakhk A. (2020). Effects of concurrent training order on follistatin, physical fitness factors and functional capacity of postmenopausal women. Med. Lab. Journa 14 (6), 28–33. 10.52547/mlj.14.6.28

[B20] Faramarz M. Bagheri L. Banitaleb E. (2018). Effect of sequence order of combined strength and endurance training on new adiposity indices in overweight elderly women. Isokinet. Exerc Sci. 26, 105–113. 10.3233/ies-172195

[B21] Ferrari R. Kruel L. F. Cadore E. L. Alberton C. L. Izquierdo M. Conceicao M. (2013). Efficiency of twice weekly concurrent training in trained elderly men. Exp. Gerontol. 48 (11), 1236–1242. 10.1016/j.exger.2013.07.016 23933066

[B22] Fyfe J. J. Bishop D. J. Stepto N. K. (2014). Interference between concurrent resistance and endurance exercise: Molecular bases and the role of individual training variables. Sports Med. 44 (6), 743–762. 10.1007/s40279-014-0162-1 24728927

[B23] García-pallarés J. Sánchez-Medina L. Carrasco L. Diaz A. Izquierdo M. (2009). Endurance and neuromuscular changes in world-class level kayakers during a periodized training cycle. Eur. J. Appl. Physiol. 106 (4), 629–638. 10.1007/s00421-009-1061-2 19396614

[B24] Glowacki S. P. Martin S. E. Maurer A. Baek W. Green J. S. Crouse S. F. (2004). Effects of resistance, endurance, and concurrent exercise on training outcomes in men. Med. Sci. Sports Exerc 36 (12), 2119–2127. 10.1249/01.mss.0000147629.74832.52 15570149

[B25] Hickson R. C. (1980). Interference of strength development by simultaneously training for strength and endurance. Eur. J. Appl. Physiol. Occup. Physiol. 45 (2-3), 255–263. 10.1007/BF00421333 7193134

[B26] Higgins J. Green S. (2008). “Chapter 8: Assessment of risk of bias in included studies,” in Cochrane Handbook for systematic reviews of interventions (Hoboken, NJ: Wiley-Blackwell: Cochrane Book Series: Cochrane Collaboration), 187–241.

[B27] Karavirta L. Häkkinen A. Sillanpää E. Hakkinen A. KauhAnen A. HaapAsAAri A. (2011). Effects of combined endurance and strength training on muscle strength, power and hypertrophy in 40-67-year-old men. Scand. J. Med. Sci. Sports 21 (3), 402–411. 10.1111/j.1600-0838.2009.01059.x 20030775

[B28] Kraemer W. J. Patton J. F. Gordon S. E. Harman E. A. Deschenes M. R. Reynolds K. (1995). Compatibility of high-intensity strength and endurance training on hormonal and skeletal muscle adaptations. J. Appl. Physiol. 78 (3), 976–989. 10.1152/jappl.1995.78.3.976 7775344

[B29] Kraemer W. J. Ratamess N. A. (2005). Hormonal responses and adaptations to resistance exercise and training. Sports Med. 35 (4), 339–361. 10.2165/00007256-200535040-00004 15831061

[B30] Lee M. J. Ballantyne J. K. Chagolla J. Hopkins W. G. Fyfe J. J. Phillips S. M. (2020). Order of same-day concurrent training influences some indices of power development, but not strength, lean mass, or aerobic fitness in healthy, moderately-active men after 9 weeks of training. PLoS One 15 (5), e0233134. 10.1371/journal.pone.0233134 32407361 PMC7224562

[B31] Makhlouf I. Castagna C. Manzi V. Laurencelle L. Behm D. G. Chaouachi A. (2016). Effect of sequencing strength and endurance training in young male soccer players. J. Strength Cond. Res. 30 (3), 841–850. 10.1519/JSC.0000000000001164 26332782

[B32] Mcbride A. Ghilagaber S. Nikolaev A. Hardie D. G. (2009). The glycogen-binding domain on the AMPK beta subunit allows the kinase to act as a glycogen sensor. Cell Metab. 9 (1), 23–34. 10.1016/j.cmet.2008.11.008 19117544 PMC2642990

[B33] Mcgawley K. Andersson P. I. (2013). The order of concurrent training does not affect soccer-related performance adaptations. Int. J. Sports Med. 34 (11), 983–990. 10.1055/s-0033-1334969 23700329

[B34] MoghadamB. H. BagheriR. Ashtary-larkyD. TinsleyG. M. EskandariM. WongA. (2020). The effects of concurrent training order on satellite cell-related markers, body composition, muscular and cardiorespiratory fitness in older men with sarcopenia. J. Nutr. Health Aging 24 (7), 796–804. 10.1007/s12603-020-1431-3 32744578

[B35] Murlasits Z. Kneffel Z. Thalib L. (2018). The physiological effects of concurrent strength and endurance training sequence: A systematic review and meta-analysis. J. Sports Sci. 36 (11), 1212–1219. 10.1080/02640414.2017.1364405 28783467

[B36] Nindl B. C. Jones B. H. Van Arsdale S. J. Kelly K. Kraemer W. J. (2016). Operational physical performance and fitness in military women: Physiological, musculoskeletal injury, and optimized physical training considerations for successfully integrating women into combat-centric military occupations. Mil. Med. 181, 50–62. 10.7205/MILMED-D-15-00382 26741902

[B37] Pinto S. S. Alaberton C. L. Cadore E. L. Zaffari P. Baroni B. M. Lanferdini F. J. (2015). Water-based concurrent training improves peak oxygen uptake, rate of force development, jump height, and neuromuscular economy in young women. J. Strength Cond. Res. 29 (7), 1846–1854. 10.1519/JSC.0000000000000820 25559906

[B38] Pinto S. S. Alberton C. L. Bagatini N. C. Zaffari P. Cadore E. L. Radaelli R. (2015). Neuromuscular adaptations to water-based concurrent training in postmenopausal women: Effects of intrasession exercise sequence. Age (Dordr). 37 (1), 9751. 10.1007/s11357-015-9751-7 25643897 PMC4315433

[B39] Pinto S. S. Cadore E. L. Alberton C. L. Zaffari P. Bagatini N. C. Baroni B. M. (2014). Effects of intra-session exercise sequence during water-based concurrent training. Int. J. Sports Med. 35 (1), 41–48. 10.1055/s-0033-1345129 23771835

[B40] Rønnestad B. R. Hansen E. A. Raastad T. (2010). Effect of heavy strength training on thigh muscle cross-sectional area, performance determinants, and performance in well-trained cyclists. Eur. J. Appl. Physiol. 108 (5), 965–975. 10.1007/s00421-009-1307-z 19960350

[B41] Rønnestad B. R. Hansen E. A. Raastad T. (2011). Strength training improves 5-min all-out performance following 185 min of cycling. Scand. J. Med. Sci. Sports 21 (2), 250–259. 10.1111/j.1600-0838.2009.01035.x 19903319

[B42] Ruiz-Alias S. A. García-Pinillos F. Jaén-Carrillo D. Perez-Castilla A. (2022). Effect of intra-session exercise sequence of an 8-week concurrent training program on the components of physical fitness in recreationally trained young adults. J. Sports Sci. 40 (15), 1722–1731. 10.1080/02640414.2022.2103615 35856521

[B43] Salamat K. M. (2017). The effect of two types of concurrent training on VO2max, maximal strength and body fat percentage in young men. Rep. Health Care, 17–22.

[B44] Schumann M. Küüsmaa M. Newton R. U. Sirparanta A. I. Syvaoja H. Hakkinen A. (2014). Fitness and lean mass increases during combined training independent of loading order. Med. Sci. Sports Exerc 46 (9), 1758–1768. 10.1249/MSS.0000000000000303 24518195

[B45] Schumann M. Ylipeltola K. Abbiss C. R. Hakkinen K. (2015). Cardiorespiratory adaptations during concurrent aerobic and strength training in men and women. PLoS One 10 (9), e0139279. 10.1371/journal.pone.0139279 26418015 PMC4587735

[B46] Shiotsu Y. Watanabe Y. Tujii S. Yanagita M. (2018). Effect of exercise order of combined aerobic and resistance training on arterial stiffness in older men. Exp. Gerontol. 111, 27–34. 10.1016/j.exger.2018.06.020 29953951

[B47] Shiotsu Y. Yanagita M. (2018). Comparisons of low-intensity versus moderate-intensity combined aerobic and resistance training on body composition, muscle strength, and functional performance in older women. Menopause 25 (6), 668–675. 10.1097/GME.0000000000001060 29406427

[B48] SillanPää E. Häkkinen A. Punnonen K. Hakkinen A. Laaksonen D. E. (2009). Effects of strength and endurance training on metabolic risk factors in healthy 40-65-year-old men. Scand. J. Med. Sci. Sports 19 (6), 885–895. 10.1111/j.1600-0838.2008.00849.x 19508653

[B49] Vikmoen O. Ellefsen S. Trøen Ø. Hollan I. HanestadhaugenM. RaasTad T. (2016). Strength training improves cycling performance, fractional utilization of VO2max and cycling economy in female cyclists. Scand. J. Med. Sci. Sports 26 (4), 384–396. 10.1111/sms.12468 25892654

[B50] Vikmoen O. Raastad T. Seynnes O. Bergstrom K. Ellefsen S. Ronnestad B. R. (2016). Effects of heavy strength training on running performance and determinants of running performance in female endurance athletes. PLoS One 11 (3), e0150799. 10.1371/journal.pone.0150799 26953893 PMC4783109

[B51] Vikmoen O. Rønnestad B. R. Ellefsen S. Raastad T. (2017). Heavy strength training improves running and cycling performance following prolonged submaximal work in well-trained female athletes. Physiol 5 (5), e13149. 10.14814/phy2.13149 PMC5350167 28292885

[B52] Wilhelm E. N. Rech A. Minozzo F. Botton C. E. Radaelli R. Teixeira B. C. (2014). Concurrent strength and endurance training exercise sequence does not affect neuromuscular adaptations in older men. Exp. Gerontol. 60, 207–214. 10.1016/j.exger.2014.11.007 25449853

[B53] Wilson J. M. Marin P. J. Rhea M. R. Wilson S. M. C. Loenneke J. P. Anderson J. C. (2012). Concurrent training: A meta-analysis examining interference of aerobic and resistance exercises. J. Strength Cond. Re 26 (8), 2293–2307. 10.1519/JSC.0b013e31823a3e2d 22002517

[B54] Yu H. J. (2014). A review of concurrent training of strength and endurance and strategies to optimize performance for athletes. China Sport Sci. 34 (02), 18–33.

