# Corrigendum: Effects of concurrent training sequence on VO2_max_ and lower limb strength performance: a systematic review and meta analysis

**DOI:** 10.3389/fphys.2023.1192593

**Published:** 2023-05-04

**Authors:** Jiuxiang Gao, Liang Yu

**Affiliations:** ^1^ Laboratory of Exercise Physiology, College of Sports Science, Beijing Sport University, Beijing, China; ^2^ Laboratory of Fitness Training, College of Fitness Training, Beijing Sport University, Beijing, China

**Keywords:** concurrent training, training sequence, endurance training, strength training, VO2_max_, lower limb strength

In the published article, there was an error in the **Funding statement**. The first grant number from the Fundamental Research Business Expenses of the Central Universities was incorrect. The incorrect grant number was 2021ZD001. The correct grant number is 2022YB013. The correct **Funding statement** appears below.

“Funding

This research was funded by a grant from the Open Project of State Key Laboratory of Basic and Applied Aerospace Medicine (SMFA20K04); Fundamental Research Business Expenses of the Central Universities (2022YB013,20221019); National Key R&D Program of the Ministry of Science and Technology (32071168); The authors report no involvement in the research by the sponsor that could have influenced the outcome of this work. The systematic review registration number PROSPERO 2019: CRD42018110290”.

The authors apologize for this error and state that this does not change the scientific conclusions of the article in any way. The original article has been updated.

